# Attacks on Health Care Workers in Historical Pandemics and COVID-19

**DOI:** 10.1017/dmp.2022.275

**Published:** 2022-12-07

**Authors:** Brett C. A. van Stekelenburg, Harald De Cauwer, Dennis G. Barten, Luc J. Mortelmans

**Affiliations:** 1 Department of Emergency Medicine, VieCuri Medical Center, Venlo, the Netherlands; 2 Department of Neurology, St. Dimpna Regional Hospital, Geel, Belgium; 3 Faculty of Medicine and Health Sciences, University of Antwerp, Wilrijk, Belgium; 4 ZNA, Camp Stuivenberg, Antwerp, Belgium; 5 Center for Research and Education in Emergency Care, University of Leuven, Leuven, Belgium; 6 Research Group on Emergency and Disaster Medicine (ReGEDiM), VUB, Brussels, Belgium

**Keywords:** COVID-19, Ebola, health care workers, pandemic, terrorism

## Abstract

Previous pandemics have been (mis)used for (geo)political reasons, for terrorism purposes, and in times of conflict. Coronavirus disease (COVID-19) has been no exception with populist politicians challenging the relations with China, calling it the “Chinese virus,” certain state actors setting up cyberterrorist actions against health care organizations in the United States and Europe, and a reported increase of violent acts against health care workers.

Aside from state-driven factors, both left- and right-wing activists and anti-vaccination activists adhering to conspiracy theories are a threat for health care organizations. Furthermore, socioeconomic, religious, and cultural factors play a role in why health care is a possible target of violence. Fear of viral pathogens, fury about financial losses due to the pandemic and governmental measures such as lockdowns, anger because of mandatory quarantines, and the disruption of burial rituals are among the reasons for people to revolt against health care providers.

Here, we provide a narrative review of the impact of violence against health care workers during the COVID-19 pandemic and earlier pandemics, and suggest preventive strategies.

During the first wave of the coronavirus disease (COVID-19) pandemic (caused by severe acute respiratory syndrome coronavirus 2 [SARS-CoV-2]) in 2020, citizens were applauding health care workers (HCWs) in the frontline response for their commitment and care. Although many people support them, HCWs face threats of various kinds.^1^ An international study showed that HCWs from 173 countries experienced more COVID-19-related stigma than other citizens.^2^ Taylor and co-workers blamed the “COVID stress syndrome” for the stigma of HCWs. They found that stigmatization attitudes toward HCWs were unrelated to altruistic support (eg, applauding) and were associated with the tendency to stay at home and to avoid other people, drugstores, and supermarkets.^3^

As the pandemic emerged, both right-wing- and left-wing-inspired organizations and anti-vaccination activists met on the Internet and spread fake news seasoned with anti-government and anti-mainstream health care narratives.^4–6^

During a pandemic, HCWs are deployed in the front line. They are the canaries in the coal mine sounding the alarm that danger is imminent, they are on the barricades struggling to contain the outbreak, they are searching for possible treatments and vaccines, they are the piano player (standing in front of politicians) trying to explain the severity of the outbreak and trying to avoid health care system collapse, and they bring bad news to families when another casualty of the war against COVID-19 has to be deplored. These are just a few of the reasons HCWs are facing threats and anger of a population in distress.

Here, we provide a narrative review of the impact of violence against HCWs during the current COVID-19 pandemic and historical pandemics, and suggest preventive strategies.

## Methods

This was a narrative overview of the current literature about violence against HCWs during pandemics. Literature was retrieved through public search engines (PubMed, Medline, Google Scholar, Google News), authoritative texts, and hand searches of references in relevant publications. Scientific studies and gray literature reports were only included if they were published in full and in the English or Dutch language. The search was last updated on February 14, 2022. Search terms included, but were not limited to: “pandemic,” “epidemic,” “health care workers,” “terrorism,” “Ebola,” “plague,” “black death,” “HIV,” “AIDS,” “polio,” “measles,” “COVID-19,” “coronavirus,” “violence,” “extremists,” and their synonyms. Exclusion criteria included articles that did not discuss the key terms as listed; however, there was no exclusion for method of publication.

In addition to the literature review, the Global Terrorism Database (GTD) was searched using the search terms, “Ebola,” “polio,” “measles,” “HIV,” “AIDS,” “dengue,” and “vaccines.” The GTD is an open-source database containing over 200 000 global terrorism incidents that occurred in the period from January 1970 through December 2019. The year 2020 was not yet available at the time of the search. The GTD is maintained by the National Consortium for the Study of Terrorism and Responses to Terrorism (START) at the University of Maryland, USA, and is part of the US Department of Homeland Security’s (Washington, DC, USA) Centre of Excellence.^7^ The GTD defines a terrorist attack as “the threatened or actual use of illegal force and violence by a non-state actor to attain a political, economic, religious, or social goal through fear, coercion, or intimidation.”

## The Factors Involved in Hostility and Violence Against HCWs

### Fear

The governments’ pandemic contingency plans, quarantine policies, and lockdown measures may lead to enhanced fear of income loss and losing jobs. This is certainly the case in diseases that are not yet well known and where a cure or vaccine is still unavailable. When the plague disrupted Italy in the 16th century, “cities like Palermo, Milan, Padua, and Venice recognized the difficulties that quarantine and blockade imposed on commerce and the employment of artisans and labourers.”^8^ Mutatis mutandis, just the same, is happening in managing the COVID-19 pandemic: Governments are struggling to find the best way to deal with the pandemic without negatively affecting the economy and civil rights.

Inaccurate information of the government can play a role in the distrust of citizens toward HCWs. This is especially the case when health care lacks resources (eg, insufficient available intensive care beds) or if knowledge about the novel pathogen is lacking, notwithstanding government officials proclaiming that the pandemic is completely under control.^9^

Fear for the unknown can lead to civil unrest, stigmatizing patients and HCWs, ultimately leading to attacks against HCWs or hospitals. This was demonstrated during earlier epidemics like Ebola in Africa and SARS in Hong Kong, and the hosting of refugees in Western Europe.^7,10^

L’ Histoire se répète. Even historical pandemics like the Black Death (14th century) and cholera (19th century) were defaced with riots and violence against HCWs, quarantine buildings and hospitals, in both America and Europe.^8^

During a pandemic, HCWs face many stress factors, such as fear of contracting the disease themselves and fear of passing the disease on to their beloved ones. Both the patients and HCWs can be considered as possible sources of infection.^11^ This was also the case with HCWs returning home from a mission abroad to fight Ebola.^12^ In 2020, HCWs in Mexico were refused access to public transport, and nurses and doctors were pelted with eggs and physically assaulted. Similar attacks were reported in the Philippines, India, the United States, and Australia.^13,14^ In January 2020, Hong Kong hotels/hospitals predestined for the reception of SARS-CoV-2-infected patients were besieged for fear of contagion of residents.^15^ In India and Cameroon, HCWs were harassed by patients or their relatives, not accepting the positive swab results and enforced quarantine.^16–18^ Similar unrest happened in Nigeria where, after a strike for 2 weeks, nurses returned to work only when armed guards were posted around the clock.^19^ Protesters in Abidjan, Ivory Coast, destroyed a COVID-19 center because of fears that HCWs might spread the disease.^20^

Even outside their work setting, HCWs are confronted with COVID-19-related stigma. There are numerous examples of HCWs being assaulted or evicted by fearful landlords in the United Kingdom, United States, Pakistan, India, Myanmar, and the Philippines.^1,14,20–22^ In Italy, there was a fatal case of domestic violence. A medical student was killed by her partner who falsely accused her of exposing him to SARS-CoV-2.^1^

#### Suggested preventive strategies

- States should investigate whether the rights of HCWs are adequately protected. These include access to safe and adequate working conditions and the right to freedom of speech.^9^
- The health care system and governments need a multimodal strategy to reach out to people and to limit fear. An increase in trust of governments might reduce global infections.^23^
- A consistent and reliable communication strategy from the government, health care systems, and experts in the field is necessary to limit the spread of fake news and conspiracy theories. Education in recognizing fake news, community engagement strategies, and cultivating empathy and confidence by providing news conferences may enhance adherence to recommendations.^24–26^
- Governments and health care organizations can apply the World Health Organization’s (WHO) COVID-19 risk communication package for health care facilities, as well as a guide for governments, the media, and local organizations to prevent and address social stigma.^13^
- Furthermore, health professional associations and (para)medical societies should unite in speaking out and condemning all acts of discrimination, intimidation, and violence against HCWs.^22,27^

### Religious and Sociocultural Motives

In Cameroon, the remains of COVID-19 deceased persons were exhumed by relatives to give them a “proper” burial.^17^ Earlier, in Sierre Leone during the Ebola virus disease epidemic, unsafe (washing the body of the deceased) burial practices became a route of Ebola virus transmission. There were not enough trained burial teams to manage the number of reported deaths.^28^

Some ultra-conservative religion groups tend to reject vaccines more often^29^ while being exposed to a higher risk for COVID-19 due to the intimacy of their religious practice.^30^ A study by Corcoran et al. found that Christian nationalism in the United States is one of the strongest predictors of COVID-19 vaccine hesitancy and is negatively associated with vaccine uptake.^31^

Many religious communities did move to online services to stay in contact with their followers during the COVID-19 pandemic,^32^ but some conservative or fundamentalist groups continued their on-site services. This happened, for example, in the Bible Belt region in the Netherlands.^33^ Of note, these services were provided without violence against HCWs.

#### Suggested preventive strategies

- Reaching out and the development and local training of a national standard operating procedure for safe, dignified burials and the deployment of additional burial teams with adequate resources (eg, personal protective equipment, other equipment, body bags, and shovels) increased community support for these practices.^28^
- The community approach and the active involvement of religious leaders increase engagement and adherence to contingency rules in isolated religious groups.^30,31,34^ Therefore, continuing religious congregations during a lockdown in adjusted form (online instead of on-site) may help increase awareness, spread information, and reduce anger and/or fear against HCWs.

### Governments Acting Against Whistleblowers, HCWs, and Scientists

In China, Malaysia, the United States, Russia, and Egypt, HCWs were fired or even imprisoned because they demanded personal protective equipment or launched critical notes via social media. Dr Li Wenliang (1986-2020) was the most prominent whistleblower in the Chinese COVID-19 epidemic and was initially silenced and humiliated by the government.^9,35^ The Brazilian president fired his health minister for speaking in favor of anti-pandemic measures.^36^ The Belgian former Minister of Health declared in February 2021 that “the experts are now announcing a third, fourth and fifth wave. The population is disgusted by this information and communication, which does not offer any perspective.”^37^

Much of the same rhetoric was used by the former US president when he addressed his supporters: “People are tired of COVID. I have these huge rallies. Just leave us alone. People are tired of hearing Fauci and all these idiots.” Anthony Fauci, the key advisor in the US response to COVID-19, received death threats accusing him of contradicting the president and politicizing the response. The turbulent relationship with the former president played an essential role in this dynamic.^1,38^

Williams and co-workers conclude that these politicians, and other populist political leaders, denied the catastrophic impact of the COVID-19 pandemic, censored HCWs and scientists when speaking about the pandemic and government failures, and decimated the national COVID-19 response.^36^

#### Suggested preventive strategies

- Amnesty International is concerned about states that jeopardize the rights of HCWs and the freedom of speech. Supranational courts (eg, European Court of Human Rights) should guarantee safety and freedom of HCWs.^9^
- As in other countries, European governments must substantiate whether the extraordinary measures taken to prevent the further spread of a disease are adequate and proportionate to the situation.
- Furthermore, they are required to keep the Council of Europe fully informed of the measures that they have taken, as well as when such measures have ceased to operate (Art. 15(3) of the European Convention of Human Rights).^39^ A simple tool to assess basic human rights of COVID-19 measures was recently presented by 2 scientists of University of Oslo, Norway.^40^
- To intensify the global response to COVID-19, the WHO might need more financial and executive resources.^36^
- Concerning the conservation of the rights and safety of HCWs, India and the UK are passing new model legislation against assaults on HCWs.^1,21^

### Ethnical Considerations, Racism, Hatred

The right-wing extremist narrative (also see the following section) mixes racist motives, apocalyptic thinking, and conspiracy theories (eg, anti-vaccination and anti-5G campaigns). In 2020 and 2021, the right-wing narrative was fueled by the QAnon movement and the former US president who repeatedly called SARS-CoV-2 the “Chinese Virus.” In Italy, it was reported that Chinese restaurants were avoided and that parents were reluctant to send their children to class if they had a classmate of Chinese origin.^1,41–43^ In the same narrative on right-wing inspired blogs, Israel and others were regarded as the scapegoat for creating SARS-CoV-2 and asylum seekers and immigrants (especially Jews and Muslims) for wanting to spread the virus.^41–43^ Another phenomenon during the COVID-19 pandemic is the deliberate spread of misinformation by Russian cyber-propaganda. For example, the COVID-19 response in Russia is praised while fake news is spread about outcome figures in other countries, again with the stigmatization of certain population groups.^4^

The history of pandemics taught us that ethnical groups or specific professions were targeted in successive pandemics. The Black Death of 1347-1351 resulted in the murder of Catalans in Sicily, clerics and beggars in Narbonne and other regions, and the pogroms against Jews. In subsequent strikes of the Black Death in the 16th and 17th centuries, the anti-Jew narrative was less pronounced. Instead, HCWs were in the line of fire and were accused of perpetuating the contagion.^8^

In the 20th century, the human immunodeficiency virus (HIV) pandemic resulted in homophobia.^44^ Haitians were blamed for bringing HIV to the United States.^45^ Similarly, unsubstantiated anti-Haitian stigma during a cholera epidemic jeopardized the contingency measures.^46^ Even nowadays, anti-homosexual legislation hampers the medical care of HIV.

#### Suggested preventive strategies

- As in previous chapters, reaching out to the people and training popular opinion leaders were effective in reducing avoidance intent and HIV-related stigma and improving compliance to universal precaution. A multidisciplinary (medical, psychological, and social) approach led by health policy-makers and planners seems to be the most appropriate in managing and reducing stigma and discrimination.^47,48^

### Doom Thinking and Conspiracy Theories

Doom thinking and conspiracy theories of left-wing as well as right-wing extremism use the COVID-19 pandemic to proclaim their ideology, undermine the state, and recruit new members. Specific social media platforms acting in a gray zone are mainly used for spreading their propaganda. They aim to attack police officers, politicians, and hospitals.^4,42^ Studies have shown that, although they have different motives, both far-left and far-right ideologies are associated with a tendency to reject vaccines for COVID-19.^49^ It should be acknowledged that left-wing attacks against HCWs are generally less violent and may therefore be reported less frequently in news reports and research articles.^50^

Right-wing extremists mix racist motives, apocalyptic thinking, conspiracy theories, and anti-science and anti-vaccination theories. Denying the pandemic, non-adherence to lockdown measures, and declining vaccination could hamper the COVID-19 response.^51^ Furthermore, subgroups believe that the government wants to control the people with the global rollout of the 5G network and to track people by inserting microchips via vaccinations.^4,42^

Conspiracy theories inspire so-called lone wolves and may lead to social unrest and protests, resulting in assaults against HCWs and attacks against health care facilities. In the Netherlands, several COVID-19 testing and vaccination facilities were targeted.^52,53^ Attacks included the ignition of an explosive device, arson attacks, harassments, and vandalism. In January 2021, protesters tried to enter an emergency department and maternity hospital and torched COVID-19 testing centers during riots against COVID-19 curfew in the Netherlands.^54^

Some lone wolves took it a step further. In Michigan, United States, a man was convicted of spitting on HCWs in 2 hospitals, trying to infect them with SARS-CoV-2.^55^ In 2020, another man from Michigan, United States, was planning to steal a helicopter and use it to fire on a hospital to “liberate” COVID-19 patients.^56^ In April 2021, a train engineer intentionally crashed his locomotive at high speed near the USNS Mercy hospital ship in Los Angeles in an attempt to expose “an alternate purpose related to COVID-19 or a government takeover.”^57^ The engineer was later convicted for terrorism charges. In Missouri, United States, a man planning an attack on a school with African Americans, a mosque, a synagogue, and preparing a bombing of a hospital was shot by the Federal Bureau of Investigation, after resisting arrest.^58^ In Denmark, a vaccination center in Silkeborg was targeted in an attempted arson. Earlier, firebombs were thrown at a test center in Ballerup, north of Copenhagen.^59^ In Belgium, May 2021, a manhunt was started after a soldier with far-right ideas uttered threats to the armed forces, the government, a mosque, and virologists in a goodbye letter to his wife. He took several heavy arms from the army barracks with him and was seen posting at the apartment of a Belgian main virologist, and then disappeared. The soldier’s body was found in June 2021, and suicide was assumed to be the cause of death.^60^

#### Suggested preventive strategies

- Counter-terrorism protection measures must be provided around test centers and vaccination centers to safeguard the HCWs and patients involved. During lockdown measures, medical facilities have become the only soft targets still 24/7-accessible and thus the only soft target for terrorist attacks.^42,61^
- We recommend providing access barriers (against ram-raid or car bombings) at the main hospital and emergency department entrances and at testing/vaccination centers. Special emphasis on the protection of HCWs and health care organizations and on rapid law enforcement is mandatory.^42,53,59,62^
- Su et al. discuss the possible crisis communication solutions that health care organizations, government agencies, and media can adopt to mitigate the negative influences of COVID-19-related news on the ongoing pandemic. Timely, credible, and transparent communication is essential.^6^
- Countermeasures against fake news include fact check columns in the media, the suspension of social media accounts that continually spread misinformation, and the removal of misleading content.^6,63^
- The Terrorist Radicalization Assessment Protocol (TRAP-18) can be used in the threat risk assessment of lone actors or terrorist faction members.^64,65^

### Anti-Government and Anti-Foreigner Involvement

HCWs working for non-governmental organizations (NGOs), such as Médecins Sans Frontières (MSF), generally face more threats than regular HCWs, mainly because they work in countries facing internal conflicts. These humanitarian-aid organizations are neutral, which is not always recognized by local communities. Some states jeopardize NGOs by mixing foreign policy and humanitarian aid. In 2004, during the “War on Terrorism,” the United States sent military troops to Afghanistan, calling it a humanitarian mission. A Taliban spokesperson justified the murder of 5 MSF staff members: “Organizations like MSF work for American interests and are therefore targets for us.” If military organizations operate under a humanitarian banner, HCWs of NGOs are at great risk of being attacked. Eventually, MSF was forced to evacuate their teams from Afghanistan.^66^

NGOs play an important role in the fight against pandemics and epidemics, with the fight against Ebola being one of the most important challenges of the past decade. According to the WHO, 85 HCWs have been wounded or killed in 42 attacks on health facilities during the Kivu Ebola epidemic since January 2019. Up until March 2021, there have been 445 Ebola-related incidents globally. In these incidents, 50 health facilities were damaged and 28 HCWs killed and 72 injured. The vast majority took place in the Democratic Republic of Congo.^67^ Furthermore, the Global Terrorism Database (GTD) lists 24 terrorist attacks (resulting in 23 people killed and another 16 injured) on treatment facilities during the Kivu Ebola epidemic until December 31, 2019.^7^ In February 2019, the attacks started with 2 major attacks on Ebola treatment centers in Butembo and Katwa. A community radio broadcaster was stabbed to death in his home. He had been working with the Ministry of Health broadcasting information about Ebola. Subsequently, the treatment centers and the radio station were closed.^7,68^

A study by Vinck et al. showed that of the 961 participating adults in North Kivu, 25.5% did not believe the Ebola outbreak was real. Low institutional trust and belief in misinformation were associated with a decreased likelihood of adopting preventive behaviors, including acceptance of Ebola vaccines and seeking formal health care. These factors increase the risk of the spread of the virus.^69^ Misinformation played a significant role in this mistrust. The Harvard Humanitarian Initiative analyzed more than 80 000 WhatsApp messages sent across 8 large group chats between August 28 and October 1, 2018, in Congo. They found that 13%—some 10 400 messages—either referenced or spread rumors and misinformation about Ebola. Combatting misinformation became a key element in the fight against Ebola in North Kivu.^68^ Furthermore, a military conflict in the region that had begun in January 2015 also hindered treatment and prevention efforts due to armed conflicts. Not surprisingly, 70% of the Ebola-related attacks found in the GTD were attributed to militia groups.^7^ Also, due to this ongoing conflict, people were already distrustful of the government.

Other pandemic-related terrorist attacks that are listed in the GTD were an armed assault at government troops conducting an anti-dengue fever advocacy campaign in the Philippines, an armed assault at police officers escorting a measles vaccination team in Pakistan, and a rocket launch at a Center for Prevention of Malaria and Leishmaniasis in Afghanistan.^7^ In 2014, most HIV/AIDS health centers in Kenya had to be closed down due to the fear of attacks related to new anti-gay legislation in their neighboring country, Uganda.^44,70^

The GTD lists 169 attacks on HCWs or police officers related to polio vaccination teams or centers, starting as early as 1999 to December 2019.^7^ Most of these attacks were claimed by Tehrik-e Taliban Pakistan, a militant group claiming the polio vaccine is a Western conspiracy to sterilize children.^71^

The COVID-19 pandemic also highlights the threats that HCWs face. The International Committee of the Red Cross (ICRC) recorded 611 violent incidents against HCWs from February to July 2020. This was about 50% higher than average. Fear of the disease, lacking basic knowledge about COVID-19, and misinformation played an important role in these attacks.^72^

#### Suggested preventive strategies

- The ICRC head of health, Dr Esperanza Martinez, sums the most important preventive strategies in his 2020 news release: “To protect healthcare staff, medical facilities and patients from violence, it is of paramount importance to disseminate accurate information regarding the origin and modes of transmission and prevention of COVID-19. HCWs, patients or specific groups must not to be stigmatized or blamed for the presence or spread of the virus. We need to strengthen our collective sense of humanity if we are to make it through this pandemic.”^72^ These resulted in a checklist for the risk stratification and implementation of measures recommended by the ICRC.^73^
- The WHO presents a shorter list of 10 lessons learned from the Ebola outbreak management, that can also be applied to address the COVID-19 pandemic (Table 1).^74^

**Table 1.** Ten lessons learned from Ebola, responses that can strengthen community engagement in the fight against COVID-19

1	Involve social scientists early in the response
2	Mobilize family leaders for surveillance, early case detection, contact identification and follow-up, and quarantine
3	Treat contact persons with dignity and the empathy they deserve
4	Communicate laboratory results promptly to the patient
5	Care for the severely ill and maintain family connections
6	Prevent stigmatization of people who recover and their families
7	Recruit local staff in the response, including local people to build the structures of the response
8	Mobilize the most resistant people in the response to overcome dissent
9	Involve grass-roots leaders in the preparation and implementation of response measures, including containment and emergency preparedness.
10	Mobilize media players and take social networks into account

Source: Anoko JN, Barry BR, Boiro H, et al. Community engagement for successful COVID-19 pandemic response: 10 lessons from Ebola outbreak responses in Africa. BMJ Global Health 2020;4:e003121. doi:10.1136/bmjgh-2020-003121.

### War Zone, Regional Conflicts, and Terrorism

We usually think of attacks on health care as something that happens in the context of war or political repression. However, the previous sections showed that incidents also happen during peacetime. The ICRC published an extensive list of attacks on HCWs.^75^

Previous studies have also revealed the vulnerability of health care facilities and HCWs to terrorist attacks. Between 1970 and 2019, there were 454 terrorist attacks against hospitals and 184 attacks against emergency medical services, causing 2381 fatalities and 4016 injured.^76,77^ The incidence of attacks increased during the latest years. In the same period, vaccinators have been targeted 133 times, most of which occurred since 2010.^78^ While the COVID-19 pandemic is a breeding ground for extremism, (local) governments have been weakened, allowing violent extremism and terrorism to regain influence.^42^ Therefore, the health care risks of terrorism remain an important concern.

War-related attacks on HCWs did not stop during the COVID-19 pandemic and were reported in Yemen, Afghanistan, Syria, the Democratic Republic of Congo, and Sudan. In these cases, the attackers are usually aiming to gain a military advantage or to deny health care to enemy forces and civilian populations.^1^ In fragile and conflict-affected countries, acts of violence during the COVID-19 pandemic have already deprived hundreds of medical services and severely hindered the response. Among others, the bombardment and destruction of a 400-bed facility in Libya further reduced the ability of health authorities and aid agencies to prepare for a full-blown epidemic.^13^ In other countries where war was already ongoing, hospitals were especially targeted by the warring factions: A maternity hospital in Kabul, Afghanistan, was attacked, as well as hospitals in Tripoli and Benghazi, Libya, where the intensive care units were destroyed.^42^

During the turmoil (May 2021) between Israel and the Palestinians, a trauma clinic of MSF was damaged, and, during the raids in Gaza, 2 doctors were killed. This happened in difficult times where hospitals of MSF and the Palestinian Red Crescent Society were already overwhelmed by COVID-19 patients, and even frontline workers had not yet been vaccinated.^79,80^

#### Suggested preventive strategies

- The ICRC’s Health Care in Danger initiative has developed a strategy to protect health care from attacks in situations of armed conflict. They provide a checklist in preventing, reducing, and mitigating violence against HCWs.^73^
- Efforts to reduce violence in health care facilities do work. The ICRC partnered with hospital administrators in a South Asian country to reduce the number of guns carried into the emergency room. After 5 months of the program’s implementation, the number of guns intercepted before being brought into the ward increased from 2 to 42 per month, reducing risk for staff and patients.^75^
- On May 3, 2016, the UN Security Council implemented Resolution 2286, supported by 80 states, including steps these states could take for the protection of health care during conflicts, but, in the end, implementation of this resolution was weak.^81^
- Health care organizations are potential soft targets for terrorism. Therefore, it is of pivotal importance that these organizations increase their preparedness and resilience to threats of terrorism and violence. They should be aware of the possibilities to harden hospital defenses, so that these measures can be implemented in risk areas, during periods of conflict or when there is a terrorist threat in the vicinity of the hospital.^61,76^

**Figure 1.**
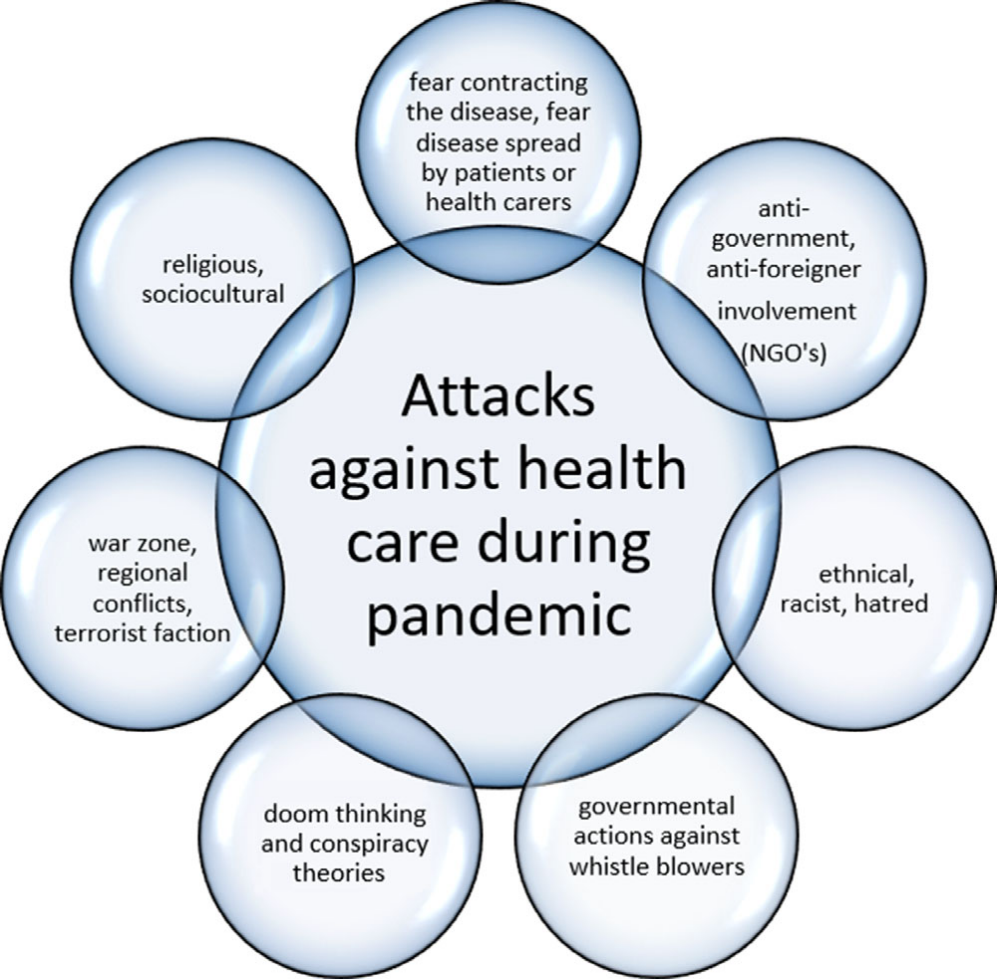
Attacks against health care during pandemic.

These factors are summarized in Figure 1.

## Discussion

During the COVID-19 pandemic, violence and threats against HCWs have reached an unprecedented high. Multiple factors play a role in this uprise, notably a mixture of fear, stress, sociocultural influences, economic crisis, religious inspiration, poverty, racism, doomsday thinking, anti-government feelings, and being fed up with the restrictions of lockdown measures. War- and conflict-related attacks on HCWs did not stop as well during the global pandemic. (see Figure 1)

On the one hand, fake news can flourish in populations that are highly resistant to governments and have no access to official information, especially in unregulated social media. Through these havens of “free speech,” an anti-science and anti-vaccination message is being spread. These platforms call their supporters to “rescue” patients from hospitals and mainstream medicine. On the other hand, reaching out to people is hampered by quarantine measures and hospital contingency plans. The patients cannot be visited by their relatives. As in the past, there is a great distance between the caregivers and the patients and their families, and the proper assistance of families in palliative settings is more difficult. This again creates a gap between HCWs and patients/relatives, leading to a higher likelihood of conflicts and violence.

History repeating itself is a common warning in all walks of life but especially in managing the current global pandemic. We witnessed similar responses of populist politicians, fearful populations in distress, stigmatization of minorities, and HCWs and NGOs being targeted – as early as the Middle Ages until now.^8,42^

### Limitations

Literature on this topic is limited in medical journals. Counter-terrorism medicine (CTM) is a novel subdiscipline in disaster and emergency medicine, and research on this topic only gained interest recently. Another limitation is that much of the fake news, conspiracy theories, and threats against scientist and HCWs are not always publicly available and could therefore not be retrieved from our search strategy. Furthermore, these conspiracies rapidly evolve as the pandemic continues. We contacted both Belgian and Dutch intelligence services, but they did not want to unravel their knowledge for national security reasons, so we could rely on only their public reports.^4,5^ Further research is warranted as there is little information on the mitigation, preparedness, response, and recovery activities in CTM.^42^

## Conclusion

Well-intended recommendations of aid organizations and supranational legislative initiatives generally do not prevent attacks on HCWs. These HCWs are the canaries in the coal mine alarming everyone that danger is imminent and the frontline workers serving on the barricades struggling to contain the outbreak and to prevent health care system collapse. They need to be protected and given full support in administering aid to so many. Medical history teaching is essential to prepare HCWs and health officials in the management of future pandemics.^8,82,83^
